# Integrated transcriptome, GWAS, and metabolome revealed the mechanism of seed germination in sorghum

**DOI:** 10.3389/fpls.2025.1601899

**Published:** 2025-07-17

**Authors:** Lan Ju, Ruizhen Liu, Xiaoqiang Cheng, Yao Wang, Xin Lv, Jianqiang Chu, Hao Niu, Haisheng Yan, Yubin Wang, Fangfang Fan, Junai Ping

**Affiliations:** ^1^ Sorghum Research Institute of Shanxi Agricultural University, Jinzhong, China; ^2^ Hou Ji Laboratory in Shanxi Province, Taiyuan, China; ^3^ College of Agriculture, Shanxi Agricultural University, Taigu, China

**Keywords:** sorghum, seed germination, transcriptome, metabolome, genome-wide association studies

## Abstract

**Introduction:**

In sorghum production, pre-harvest sprouting (PHS) is one of the most important problems, and the primary cause of sprouting susceptibility is a low dormancy prior to crop harvest.

**Methods:**

To cope with this situation, we conducted transcriptome, metabolome, and genome-wide association studies (GWAS) to understand the mechanism underlying sorghum seed dormancy and germination.

**Results:**

We constructed 36 transcriptome libraries from four sorghum materials with contrasting germination abilities at three developmental stages. The Kyoto Encyclopedia of Genes and Genomes (KEGG) analysis based on transcriptome data showed that metabolic pathways, biosynthesis of secondary metabolites, starch and sucrose metabolism, and plant hormone signal transduction are greatly enriched. In plant hormone signal transduction, genes associated with abscisic acid (ABA), gibberellic acid (GA), brassinosteroid (BR), and the auxin signaling pathway are involved in seed germination. GWAS of the 24-h germination rate across 232 cultivars identified four significant SNPs and 31 candidate genes, with *SbPP2C33* emerging as the top candidate based on transcriptome integration. Combining transcriptome and metabolome analyses revealed that genes facilitating starch/sucrose conversion to glucose, fructose, and maltose were upregulated in low-dormancy genotypes, consistent with the accumulation levels of corresponding metabolites.

**Discussion:**

In summary, our findings demonstrate that ABA signaling, mediated by *SbPP2C33*, coordinates carbohydrate mobilization during seed germination in sorghum. These findings provide novel mechanistic insights into the hormonal regulation of metabolic processes in cereal crops.

## Introduction

Pre-harvest sprouting (PHS), a term mainly used to describe the phenomenon in which grains germinate prematurely on the spike typically induced by continuous rainy and humid weather during the late maturation stage, is one of the most important problems in sorghum [*Sorghum bicolor* (L.) Moench] production in China, Argentina, and other parts of the world (Benech-Arnold and Rodríguez, 2018). This situation leads to reduced seed viability and hydrolysis of starch in the endosperm, which results in reduced grain weight and creates a favorable environment for saprophytic fungi (Lijavetzky et al., 2000). Thus, obtaining resistance to PHS is one of the main objectives in sorghum breeding programs. As in other cereals, the primary cause for sprouting susceptibility is an unbalanced dormancy/germination ratio (Tai et al., 2024).

Seed dormancy and germination are an integral stage of crop production, directly affecting crop yield and grain quality. In order to ensure rapid synchronous germination after sowing, grains with higher germination rates are a typical feature of domestication with the development of the agricultural industry (Shu et al., 2016; Henry et al., 2018). However, a certain degree of dormancy is critical for seed development and for preventing PHS in crops, which causes substantial losses in yield and quality in agricultural production (Simsek et al., 2014). Therefore, regulating seed germination to achieve appropriate dormancy in sorghum is crucial for enhancing both crop yield and quality.

The process of seed germination is complex and highly regulated, influenced by multiple factors including plant hormones, metabolite storage in seeds, and environmental factors (Tognacca and Botto, 2021). The phytohormones gibberellic acid (GA) and abscisic acid (ABA) play crucial and antagonistic roles in regulating seed germination. The balance between ABA and GA determines whether seeds germinate. ABA inhibits seed germination through ABA metabolism and the ABA core signaling pathway (PYR/PYLs-PP2C-SNRK-ABI3/4/5), which consists of ABA receptors (PYR/PYLs), protein phosphatases 2C (PP2Cs), SNF1-related protein kinases 2 (SnRK2s), and downstream transcription factors (ABI3/4/5) (Wang et al., 2020; Chen et al., 2020). In the presence of ABA, the reduced activity of PP2Cs leads to the auto-phosphorylation of SnRK2, which, in turn, activates downstream transcription factors and further inhibits seed germination (Zhao et al., 2020). Conversely, GA promotes seed germination, and GA-deficient mutants *ga1* and *ga2* show a delay or absence of seed germination (Penfield et al., 2006). Apart from ABA and GA, additional hormones like auxin/indole-3-acetic acid (IAA), jasmonate (JA), ethylene (ET), and brassinosteroid (BR) play roles in germination (Liu et al., 2013; Ju et al., 2019; Kim et al., 2019; Sun et al., 2020; Ahammed et al., 2020).

Seed germination is a complex physiological and metabolic process that involves different metabolites, such as carbohydrates, organic acids, amino acids, and flavonoids (Wei et al., 2020; Liu et al., 2021). In cereal seeds, carbohydrates and proteins stored in the endosperm are mobilized during seed germination to provide energy and substrates for developing seedlings, and α-amylase (EC 3.2.1.1) is the major enzyme involved in the hydrolysis of starch to glucose. GAs is synthesized in the embryo and transported to the aleurone layer to induce α-amylase gene expression and α-amylase synthesis. Subsequently, the induced α-amylase hydrolyzes the stored starch, generating the soluble sugars to fuel germination (Kaneko et al., 2002).

Studies on the PHS resistance in sorghum were mainly using two lines with contrasting sprouting phenomenon IS9530 (PHS resistant) and Redlan (PHS susceptible). Early work by Lijavetzky et al. identified two significant QTLs controlling seed dormancy (Lijavetzky et al., 2000). Subsequent studies by Cantoro et al. (2016) expanded these findings through F2 and F3 segregating populations, mapping six additional seed dormancy QTLs (Cantoro et al., 2016). More recently, using a recombinant inbred line (RIL) mapping population, derived from the F3 (Redlan×IS9530 cross), a particularly significant dormancy QTL (*qDOR-9*) was discovered and validated using independent near-isogenic lines. This locus was shown to improve PHS tolerance in cultivated dw1-carrying backgrounds without altering plant height (Rodríguez et al., 2025). Additionally, the role of phytohormones in regulating dormancy and PHS susceptibility has been to elucidate using these two lines (Benech-Arnold and Rodríguez, 2018).

Here, by combining with transcriptomic, metabolomic, and genome-wide association analyses, we revealed that an ABA signaling key element, *SbPP2C33*, was the most promising candidate gene responsible for seed germination ability. ABA signaling coordinates carbohydrate mobilization during seed germination in sorghum. These findings provide mechanistic insights into the hormonal regulation of metabolic processes in cereal crops.

## Materials and methods

### Planting and phenotyping

A total of 232 diverse sorghum accessions used for genome-wide association studies (GWAS), RNA sequencing (RNA-seq), and metabolite detection were planted and harvested in Jinzhong, Shanxi Province (112°42′N and 37°36′E) from 2022 to 2023 cropping seasons. From this comprehensive collection, we selected four representative lines exhibiting contrasting seed germination phenotypes for RNA-seq analysis: Zhuyeqing (Kaiyuan), Heiwobai (Luanping), TB kangya, and Jingnong2B. The complete accession list, including geographical origins and phenotypic characteristics, is provided in **Supplementary Table S1**. Each accession was planted in a 5-m, two-row plot with 50 cm between rows. Sowing density was approximately 20 seeds/row. Field management practices, including irrigation, weed control, and fertilization, followed local conventional protocols with the following specifics: drip irrigation was applied during both the seedling and grain-filling stages; fertilization consisted of a base application of 40 kg/mu compound fertilizer (N24-P10) and 15 kg/mu foliar urea; weed control was managed by applying quinclorac during the seedling and jointing stages.

For germination tests, physiological mature sorghum seeds that were characterized by the loss of green color were harvested on different days based on their different maturity period. Then, the seeds were air-dried for 7–10 days and stored in a freezer at −20°C to keep dormancy. After all seeds were collected, 50 seeds with uniform size and shape for each accession were selected and soaked in a 9-cm-diameter Petri dish containing 6 mL of distilled water at 25°C in darkness for 8 h. Germinated grains were counted after 12, 14, 16, 18, 20, 22, 24, 36, 48, 72, and 96 h. Seeds with visible radicles were considered germinated (Ju et al., 2019).

### RNA-seq library construction, sequencing, and analysis

A total of 36 RNA-seq samples from four materials and collected at three time points (12, 24, and 48 h) after seed germination were constructed for transcriptome sequencing. The total RNA of each sample used for RNA-seq assay was extracted using CTAB (cetyltrimethylammonium bromide) (Wang and Stegemann, 2010b). After RNA extraction, a Qsep400 high-throughput biofragment analyzer was used to evaluate its integrity. Oligo (dT) magnetic beads were used to enrich eukaryotic mRNA through the binding of AT complementary pairs to the poly(A) tail of the mRNA. RNA concentration was measured using a Qubit™ 4.0 Fluorometer. Then, the mRNAs were cleaved into small fragments with fragmentation buffer and used to synthesize first-strand cDNA and second-strand cDNA. The libraries were constructed using VAHTS Universal V10 RNA-seq Library Prep Kit for Illumina (NR606).

The libraries were sequenced on the Illumina NovaSeq 6000 platform. After obtaining the raw sequences, we utilized fastp (0.23.2) to remove adapter sequences and generated clean data. Paired reads were discarded based on the following criteria: if the number of “N” bases in any sequencing read exceeded 10% of that read’s total length, or if any sequencing read contained low-quality bases (with a quality score of *Q* ≤ 20) that exceeded 50% of the read’s length. We established a minimum clean data requirement of ≥6 GB per sample to ensure sufficient sequencing depth. HISAT2 software was used to map the clean reads to the sorghum reference genome (ftp://ftp.ensemblgenomes.org/pub/plants/release-52/fasta/sorghum_bicolor/dna/). In addition, novel gene prediction was performed using StringTie (2.1.6) and gene expression levels were quantified using featureCounts (2.0.3) to calculate gene alignment statistics. Subsequently, FPKM (fragments per kilobase million) values for each gene were computed based on gene length. FPKM was used to standardized the expression level (Grabherr et al., 2013). DESeq2 (1.22.1) was used for differential gene expression analysis between two groups. DEGs (differentially expressed genes) were defined as those with a |log2Fold Change| ≥ 1 and a false discovery rate (FDR) < 0.05. In addition, functional enrichment analyses [Kyoto Encyclopedia of Genes and Genomes (KEGG) and Gene Ontology (GO)] were performed using clusterProfiler (v4.6.0) with significance defined as FDR < 0.05 (Benjamini–Hochberg correction). R language Pheatmap (1.0.8) was used for clustering analysis of the different genes in samples.

### qRT-PCR

The RNA used for qRT-PCR assay was extracted using the RNA prep Pure Plant Plus Kit (Polysaccharides & Polyphenolics-rich, Tiangen, Beijing, China). Approximately 2 μg of total RNA was used for reverse-transcribing to cDNA by the abm reverse transcriptase kit. The qRT-PCR was performed with SYBR Green Premix Pro Tag HS qPCR Kitlll (Low Rox Plus) (Accurate) on a QuantStudio 6 Flex (ABI). *SbEIF4A* was used as an internal control. Experiments were performed independently three times with similar results. All the primers used for qRT-PCR are listed in **Supplementary Table S2**.

### Whole-genome resequencing and genotyping

A total of 232 diverse sorghum germplasm resources were used for whole-genome resequencing and genotyping. All accessions of DNA were extracted using the CTAB method and sequenced on the Illumina HiSeq system (Personal Bio, Shanghai, China) (Allen et al., 2006). The clean reads were mapped to the sorghum reference genome (ftp://ftp.ncbi.nlm.nih.gov/genomes/genbank/plant/Sorghum_bicolor/latest_assembly_versions/GCA_000003195.3_Sorghum_bicolor_NCBIv3) with the bwa (0.7.12-r1039) mem program. The GATK (V 3.8) software was used to perform SNP detection in the following steps: (1) Alignments near InDel are usually unreliable and need to be realigned using known InDel. (2) SNP loci for the samples were obtained using the Unified Genotyper program with stand_call_conf set to 30 and stand_emit_conf set to 10. The SNP loci were annotated using ANNOVAR (Wang et al., 2010a).

### Population genetics analysis and GWAS

A phylogenetic tree was constructed using the FastTree (v2.1.11) software (http://www.microbesonline.org/fasttree/) with bootstrap replications of 1,000 (Price et al., 2009). Linkage disequilibrium (LD) analysis was tested with LD coefficient *D′* and *r* ^2^, and PopLDdecay (V3.42) was used to calculate *r* ^2^ between SNPs: *r* ^2^ ≥ 0.33, prompt “strong LD”. Principal component analysis (PCA) of the population was performed using GCTA (v1.94.1) software (http://www.complextraitgenomics.com/software/gcta/) (Yang et al., 2011). Using the first three principal components (PCs) as covariates, GWAS analysis was performed with the FarmCPU (fixed and random model circulating probability unification model) model from the rMVP package (Yin et al., 2021). The genes near the significant SNP locus were selected as candidate genes and annotated according to the NCBI database (https://ftp.ncbi.nlm.nih.gov/genomes/all/GCF/000/003/195/GCF_000003195.3_Sorghum_bicolor_NCBIv3). Whole-genome LD was separately calculated using TASSEL 3.0 (Bradbury et al., 2007).

### Metabolite detection and analysis

After the freeze-dried seed was ground (30 Hz, 1.5 min) to powder using a mixer mill (MM 400, Retsch), 50 mg of the sample was weighed and extracted with 1.2 mL of 70% −20°C pre-cooled aqueous methanol. The samples were subsequently centrifuged at 12,000 rpm for 3 min after vortex six times. Then, the aspirated supernatant was filtered using a microporous membrane with 0.22 μm pore size and stored in the injection vial for further analysis. The UPLC-MS/MS detection was performed on a UPLC-ESI-MS/MS system (UPLC, ExionLC™ AD, https://sciex.com.cn/) and a tandem mass spectrometry system (https://sciex.com.cn/). Metabolite quantification was accomplished by using the multiple reaction monitoring (MRM) model. Then, the above data matrices with the ion intensity of metabolites were uploaded to the MultiQuant software for statistical analyses. The significant differences in the relative metabolite content were tested by PCA and orthogonal partial least squares discriminant analysis (OPLS-DA). For two-group analysis, absolute Log2FC (fold change) ≥1 and VIP (variable importance in the projection) ≥1 was used as the standards for identifying differentially abundant metabolites. The DEGs were identified and annotated based on the KEGG compound database (http://www.kegg.jp/kegg/compound/) and KEGG pathway database (http://www.kegg.jp/kegg/pathway.html), respectively.

## Results

### Analysis of germination phenotype in four different sorghum lines

**Figure 1:**
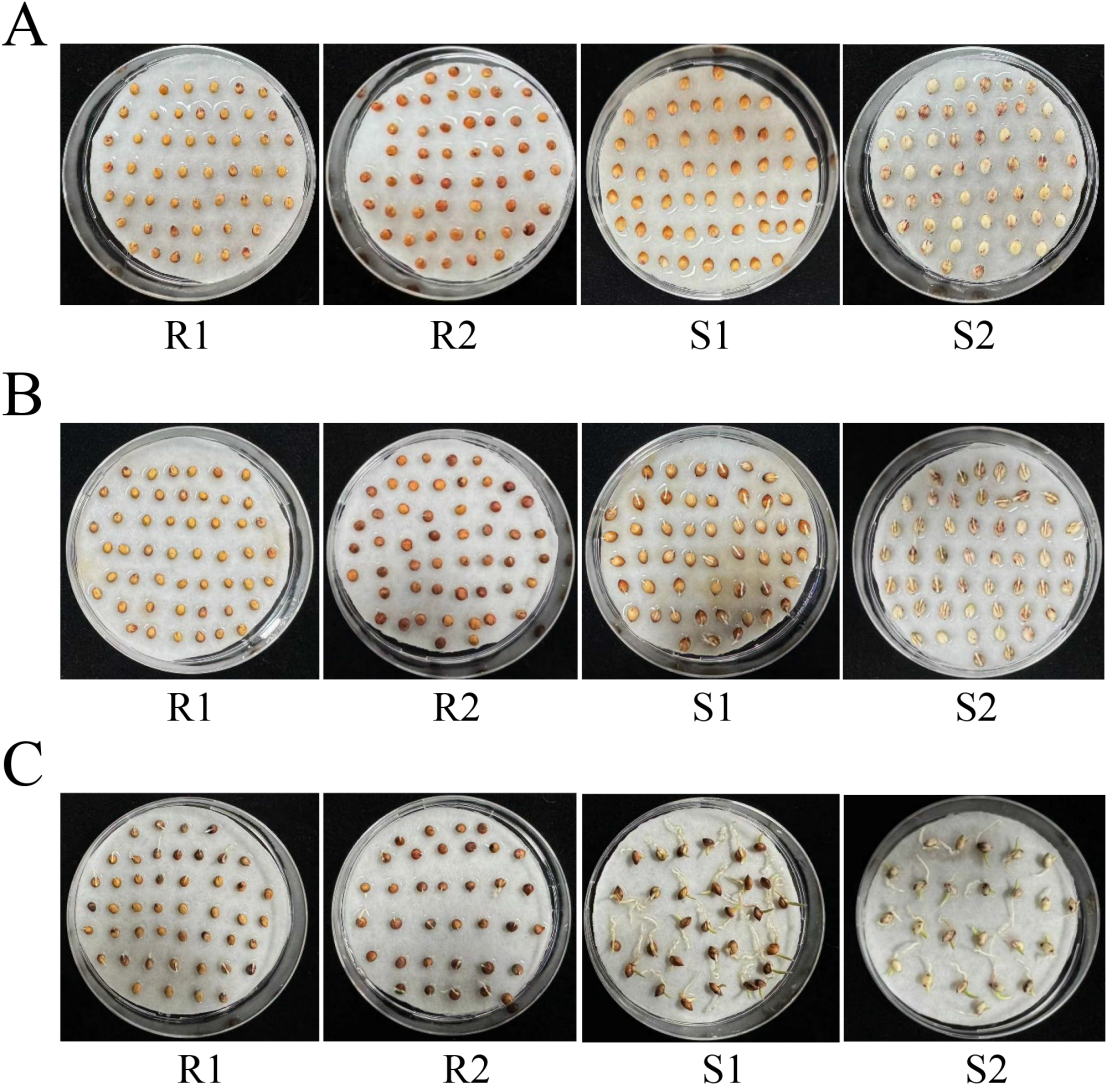
The germination phenotype varies in different sorghum lines. **(A–C)** Representative images showing the germination phenotype of four different sorghum lines at 12 h **(A)**, 24 h **(B)**, and 48 h **(C)** of seed germination. R1 and R2 represent materials with higher resistance to germination, TB kangya and Jingnong2B, respectively. S1 and S2 represent materials with lower resistance to germination, Zhuyeqing (Kaiyuan) and Heiwobai (luanping), respectively.

In this study, we selected four different sorghum lines [Zhuyeqing (Kaiyuan), Heiwobai (luanping), TB kangya, and Jingnong2B] to investigate the germination phenotype. The results showed that Zhuyeqing (Kaiyuan) and Heiwobai (luanping) showed contrasting germination abilities compared with TB kangya and Jingnong2B (**Figure 1**). At 24 h of seed germination, Zhuyeqing (Kaiyuan) and Heiwobai (luanping) showed a germination rate of 91% and 100%, respectively, while TB kangya and Jingnong2B exhibited a germination rate of only 9%–20% (**Supplementary Figure S1**). By 48 h of seed germination, the germination rate of TB kangya and Jingnong2B reached 90%–100%. We then used S1 and S2 to represent materials with lower resistance to germination, Zhuyeqing (Kaiyuan) and Heiwobai (luanping), and R1 and R2 to represent materials with higher resistance to germination, TB kangya and Jingnong2B, respectively.

### The transcriptome sequencing and gene expression analysis

We obtained raw data and clean data ranging from 41.1 to 89.6 Mbp and from 43.9 to 84.9 Mbp per library, respectively. The amount of clean data from each sample exceeded 6.16 Gb. After filtering, the Q20 percentage was greater than 97.71% and Q30 percentage was greater than 93.67%. Additionally, the GC content exceeded 53.05% (**Supplementary Table S3**). The PCA results showed that the biological replicates were clustered together, further demonstrating the reliability of the data (**Supplementary Figure S2**). Moreover, the expression patterns of four DEGs were also confirmed by qRT-PCR (**Supplementary Figure S3**, **Supplementary Table S4**).

**Figure 2:**
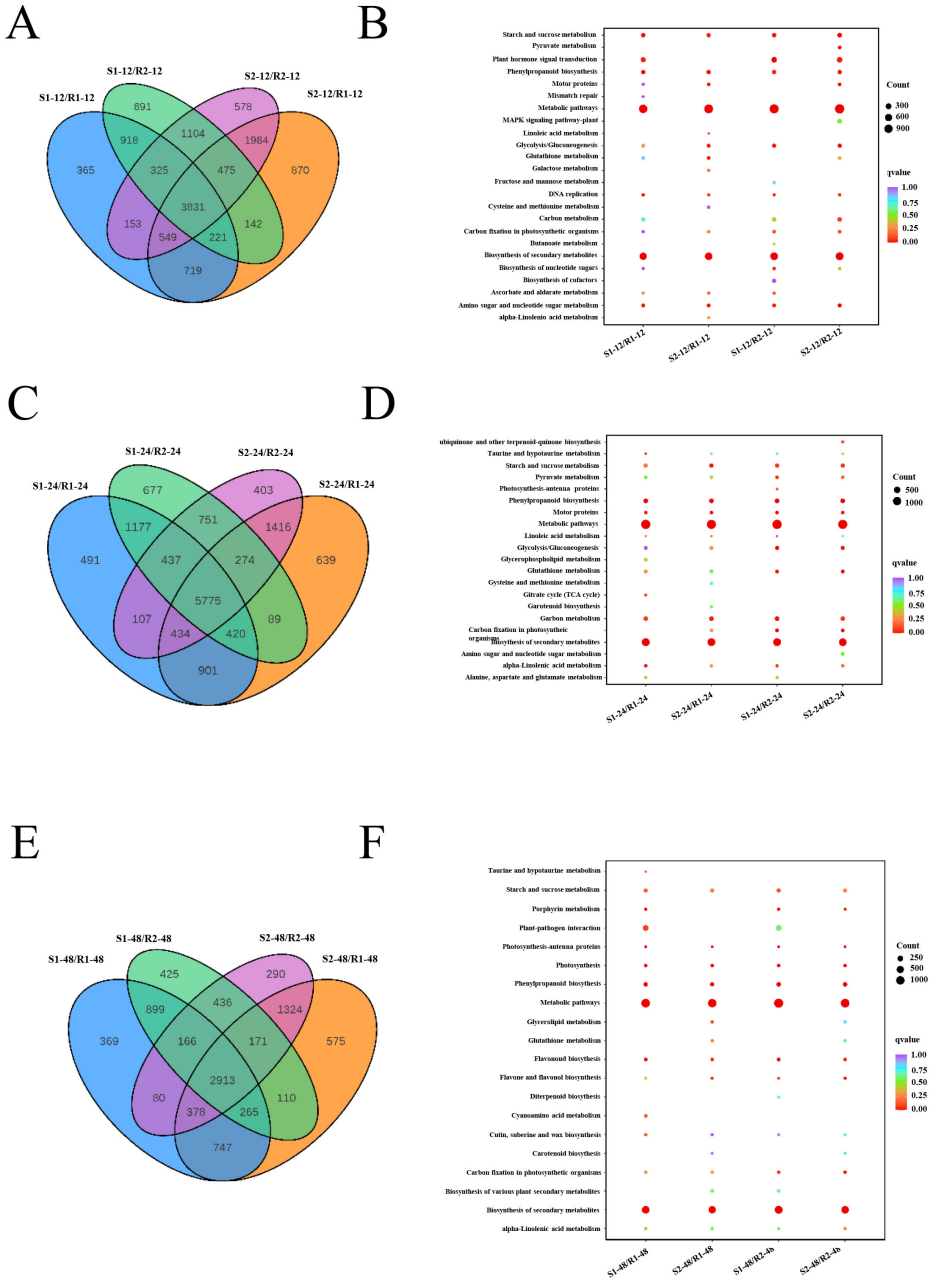
Analysis of DEGs among different samples. **(A, C, E)** Venn diagrams showing DEGs in the four different samples at 12 h **(A)**, 24 h **(C)**, and 48 h **(E)** of seed germination, respectively. **(B, D, F)** KEGG analysis of differentially expressed genes in each comparison group at 12 h **(B)**, 24 h **(D)**, and 48 h **(F)** of seed germination. R1 and R2 represent materials with higher resistance to germination, TB kangya and Jingnong2B, respectively. S1 and S2 represent materials with lower resistance to germination, Zhuyeqing (Kaiyuan) and Heiwobai (luanping), respectively.

To study the molecular mechanisms of sorghum seed germination, we further investigated the DEGs in the different varieties at three developmental stages. At 12 h after seed germination, a total of 3,831 DEGs were commonly detected in four comparison groups (S1/R1, S1/R2, S2/R1, and S2/R2) (**Figure 2A**). KEGG enrichment analysis of all DEGs based on transcriptome data revealed that the DEGs were mainly involved in metabolic pathways, biosynthesis of secondary metabolites, starch and sucrose metabolism, and plant hormone signal transduction (**Figure 2B**). At 24 h after seed germination, 5,775 DEGs were commonly detected in these four comparison groups (**Figure 2C**). KEGG analysis based on transcriptome data identified that DEGs were enriched mainly in metabolic pathways, biosynthesis of secondary metabolites, starch and sucrose metabolism, and phenylpropanoid biosynthesis (**Figure 2D**). At 48 h after seed germination, a total of 2,913 DEGs were commonly detected in these four comparison groups (**Figure 2E**). The KEGG analysis based on transcriptome data confirmed that DEGs were mainly enriched in metabolic pathways, biosynthesis of secondary metabolites, starch and sucrose metabolism, and phenylpropanoid biosynthesis (**Figure 2F**). These results indicated that metabolic pathways, biosynthesis of secondary metabolites, and starch and sucrose metabolism are commonly enriched at these three stages. Furthermore, the DEGs were subjected to GO functional analysis. At 12 h after seed germination, the significantly enriched GO terms were hydrolase activity, acting on glycosyl bonds, and hydrolase activity, hydrolyzing O-glycosyl compound. At 24 h after seed germination, the significantly enriched GO terms were reactive oxygen species metabolic process. The most enriched GO terms were photosynthetic membrane and chloroplast thylakoid membrane at 48 h (**Supplementary Figure S4**).

### Plant hormone-related DEGs in seed germination

**Figure 3:**
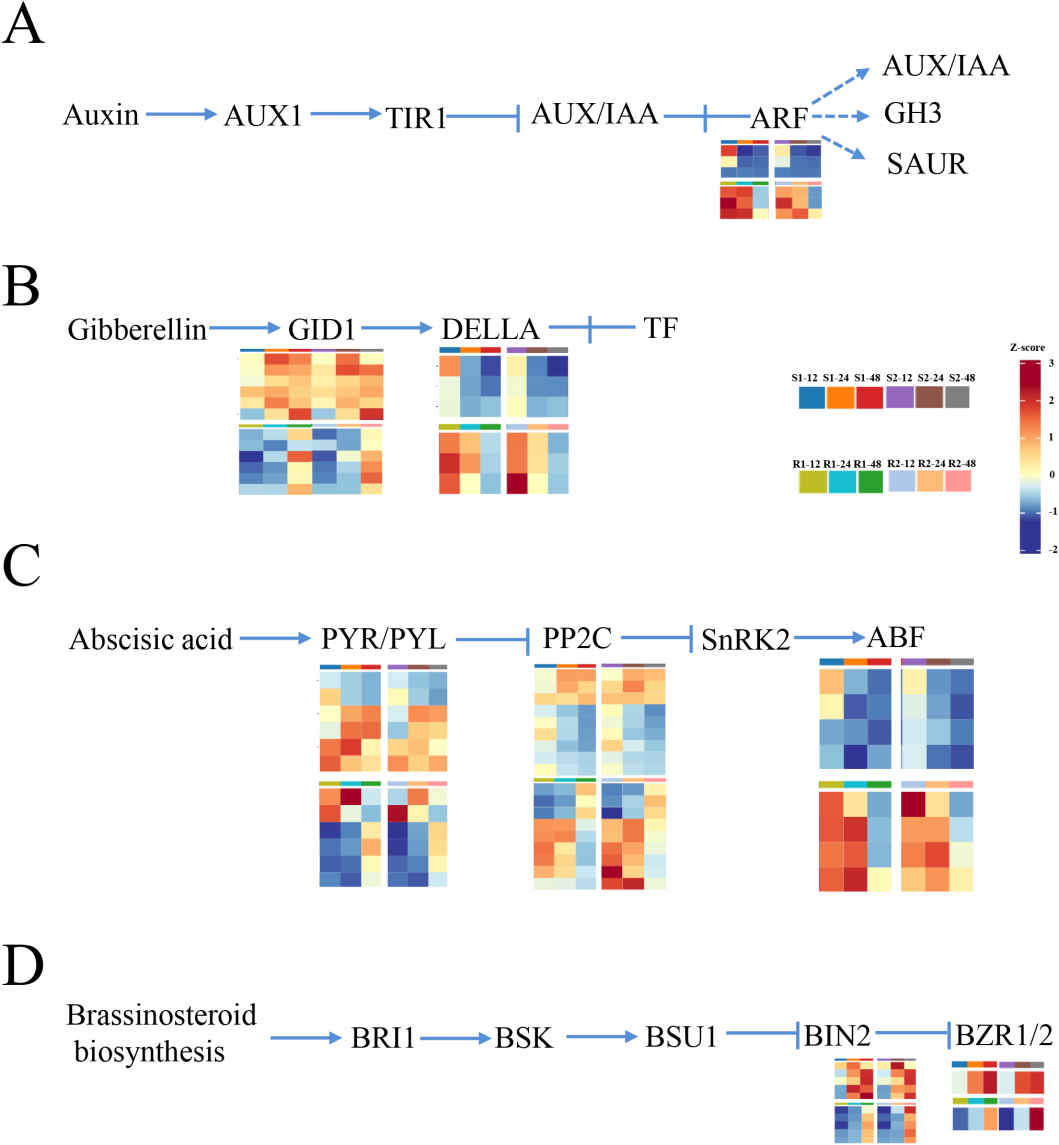
Expression pattern analysis of the hormone signaling transduction-related gene. **(A–D)** The DEGs’ expression enriched in auxin **(A)**, gibberellin **(B)**, abscisic acid **(C)**, and brassinosteroid **(D)** signaling pathways is indicated by the heatmap. R1 and R2 represent materials with higher resistance to germination, TB kangya and Jingnong2B, respectively. S1 and S2 represent materials with lower resistance to germination, Zhuyeqing (Kaiyuan) and Heiwobai (luanping), respectively.

In this study, we found that plant hormone signal transduction was greatly enriched at 12 h after seed germination, indicating that plant hormones play an important role in regulating seed germination. In the auxin synthesis regulation pathway, auxin response factor (ARF) genes were upregulated in materials with lower resistance to germination (S1 and S2) compared to materials with higher resistance to germination (R1 and R2), indicating that these genes may play positive roles in regulating seed germination (**Figure 3A**; **Supplementary Table S5**). In the GA-related pathway, gibberellin receptor GID1 genes were upregulated and DELLA protein genes were downregulated in materials with lower resistance to germination (S1 and S2) compared to materials with higher resistance to germination (R1 and R2) (**Figure 3B**; **Supplementary Table S5**). In the ABA pathway, all ABA receptor PYR/PYL (PYR/PYL) genes and three members of protein phosphatase 2C (PP2C) genes were upregulated, while six members of PP2C genes and all ABA-responsive element binding factor (ABF) genes were downregulated in materials with lower resistance to germination (S1 and S2) compared to materials with higher resistance to germination (R1 and R2) (**Figure 3C**; **Supplementary Table S5**). DEGs in the brassinosteroid biosynthesis pathway were all upregulated, including protein brassinosteroid insensitive 2 (BIN2) and brassinosteroid resistant 1/2 (BZR1/2) (**Figure 3D**; **Supplementary Table S5**). Therefore, we suggest that auxin, GA, ABA, and BR are involved in seed germination.

### GWAS for seed germination

In order to further identify candidate genes regulating sorghum seed germination, we conducted a GWAS using the whole-genome resequencing (WGRS) data of 232 sorghum germplasm resources, which include 156 from China, 30 from India, 19 from America, 13 from Africa, and 14 from other countries (**Supplementary Table S1**). In total, 13.95 billion paired-end reads were generated with sequencing depths ranging from 6.48× to 11.19×, and a genome coverage of approximately 94.01%. Following mapping against the reference genome and single-nucleotide polymorphism (SNP) calling, there were a total of 3,641,771 SNPs with minor allele frequency > 0.05 and missing rate < 10%. Based on phylogenetic tree and PCA, we clustered the 232 sorghum accessions with this SNP dataset into three clades (**Supplementary Figures S5A, B**). Clade 1 and Clade 2 contain a total of 68 varieties (lines), which are mainly wild varieties in China. Clade 3 contains 164 cultivated varieties (lines). Some of the cultivated varieties (lines) are foreign varieties from America, India, Mexico, and some African countries. Additionally, some cultivated species are from our country, which are derived from China, China/America, and China/India. The LD decay rate was measured as the chromosomal distance at which the average pairwise correlation coefficient (*r^2^*) dropped to half its maximum value. In this study, The LD decay rate was 34 kb when it decays to 20%, indicating that 34,000 bp before and after the threshold point was selected as the candidate region significantly associated with the trait (**Supplementary Figure S5C**).

**Figure 4:**
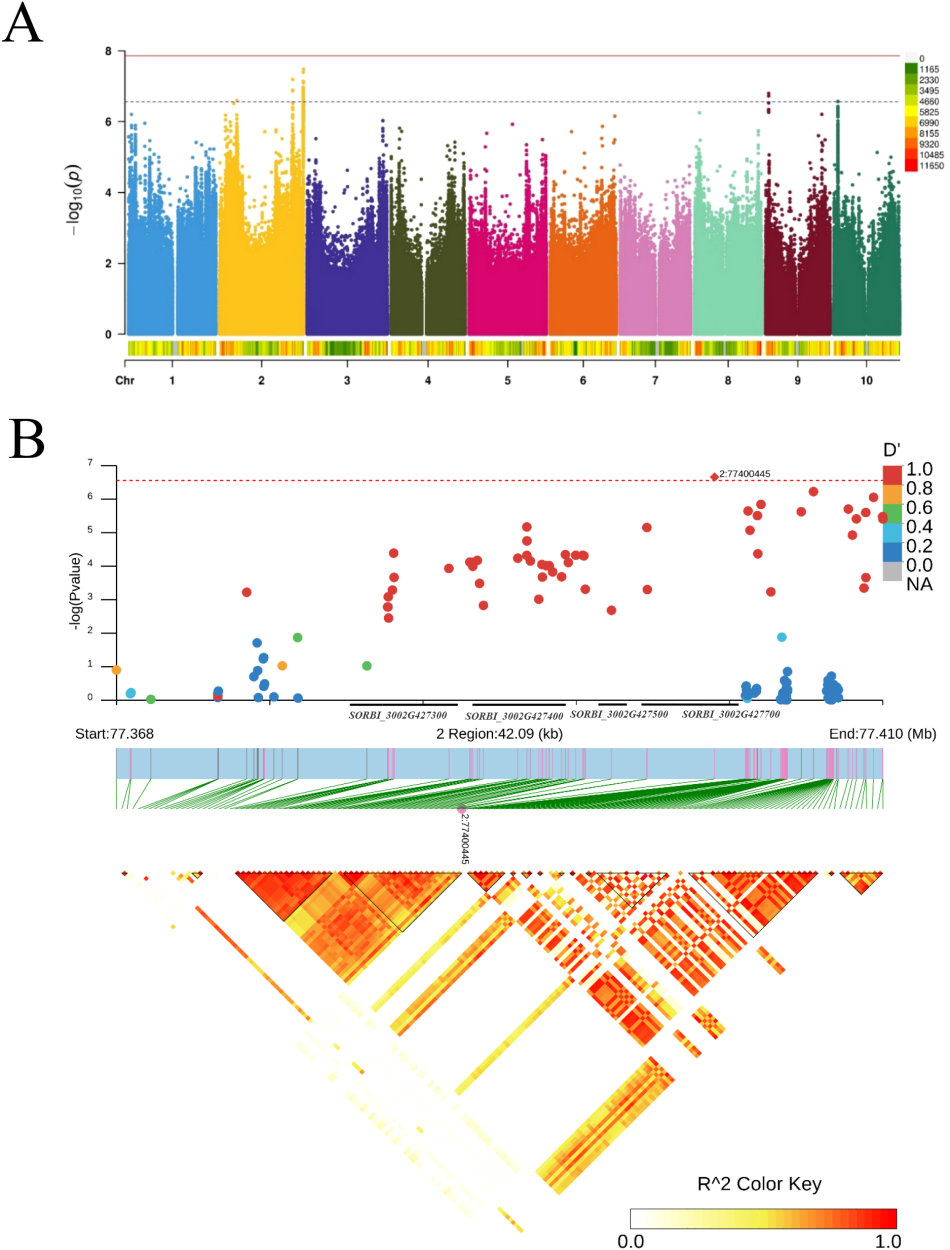
The seed germination-trait-associated loci on chromosome 2. **(A)** Manhattan plot of genome-wide association studies (GWAS) for seed germination. **(B)** Linkage disequilibrium (LD) block analysis of chr2: 77368000-77410000.

In this study, the average seed germination rate at 24 h from 2022 to 2023 was utilized to assess the seed germination ability of each sorghum line (**Supplementary Figure S6**, **Supplementary Table S6**). The trait presented abundant variations, ranging from 0 to 100% (CV = 64%). In the GWAS, FarmCPU was used to estimate marker–trait associations (Ma et al., 2020). To account for multiple testing in the genome-wide association analysis, we applied the Bonferroni correction to adjust the raw *p*-values. A total of four significant SNPs associated with seed germination rate were identified at a *p*-value threshold of 1.192hol^−6^ on chromosomes 2, 9, and 10 supported by the strong LD among associated SNPs (**Figure 4A**; **Supplementary Figure S7**). The most significant SNP was on chromosomes 2 from the 77.3 to 77.7 Mb region, including 31 candidate genes (**Figure 4B**; **Supplementary Table S7**).

### Priority candidate genes identified by integrating the GWAS and transcriptome data

We further analyzed the expression level of the 31 candidate genes detected by GWAS from the transcriptome data. Among these, six genes (*SORBI_3002G427300*, *SORBI_3002G428600, SORBI_3002G430200*, *SORBI_3002G431100, SORBI_3002G428800*, and *SORBI_3002G428800*) were differentially expressed in four comparison groups (S1/R1, S1/R2, S2/R1, and S2/R2) at three developmental stages (**Supplementary Figure S8**). These 6 genes: 1. PP2C 33 isoform X2; 2. hypothetical protein ; 3. ubiquitin-activating enzyme E1; 4. AMP deaminase; 5. DNA replication complex GINS protein PSF2; 6. L-ascorbate peroxidase 2, cytosolic (**Supplementary Table S8**).

As shown in the results, the expression levels of *SORBI_3002G427300*, *SORBI_3002G428600*, *SORBI_3002G430200*, and *SORBI_3002G431100* were upregulated in materials with lower resistance to germination (S1 and S2) compared to materials with higher resistance to germination (R1 and R2) at 12 and 24 h after seed germination; the expression level of *SORBI_3002G428800* was only upregulated in S1 and S2 compared to R1 and R2 at 12 h after seed germination only, indicating that these genes may play positive roles in regulating seed germination. However, the expression level of *SORBI_3002G429700* was downregulated in S1 and S2 compared to R1 and R2 at three developmental stages, indicating that this gene may play a negative role in regulating seed germination (**Supplementary Figure S8**).

Previous studies have shown that PP2C functions as a key negative regulator of the ABA signaling pathway. Coincidentally, from our transcriptome data, it is also a critical gene in regulating seed germination (**Figure 3**). Therefore, we assigned *SbPP2C33* (*SORBI_3002G427300*) as the most promising candidate gene responsible for the seed germination ability in this study.

### Haplotype analysis of *SbPP2C33*

**Figure 5:**
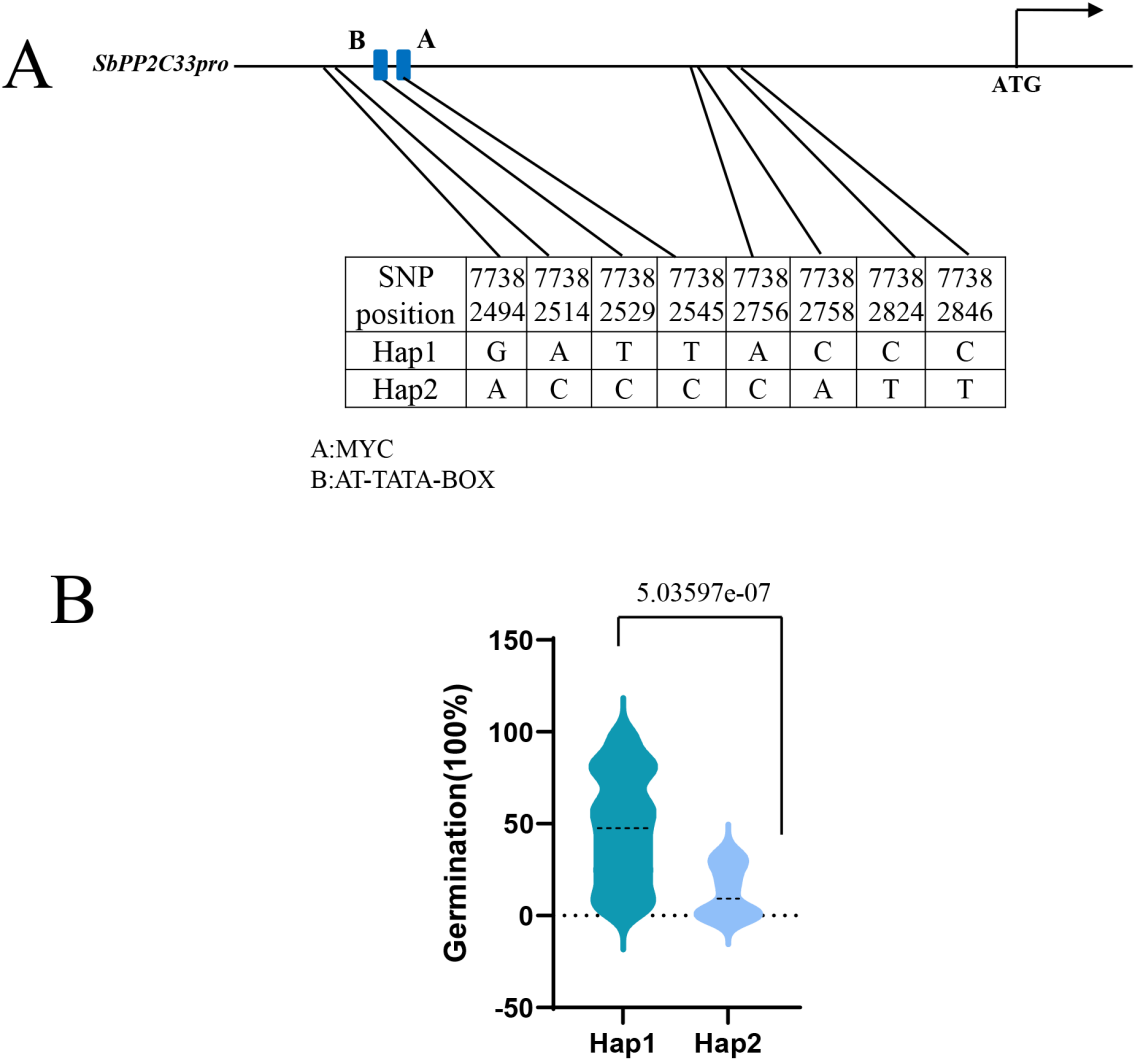
Variation of *SbPP2C33* haplotypes controlling seed germination. **(A)** Two haplotypes (Hap1 and Hap2) were determined for *SbPP2C33* based on the variations of eight SNPs. Hap, haplotype. **(B)** Haplotype analysis of *SbPP2C33*.

Moreover, the haplotype analysis of *SbPP2C33* was conducted, revealing two major haplotypes (Hap1 and Hap2) in *SbPP2C33*, comprising over 95% of the population. Hap1 was the most prevalent, comprising 213 lines, while Hap2 includes only 12 lines. Eight SNP loci determined the haplotype classification, and all of these SNPs were located in the promoter region of *SbPP2C33.* SNP77382529 and SNP77382545 were located in the MYC and AT-TATA-BOX elements, respectively (**Figure 5A**). Furthermore, we analyzed the significant differences in seed germination between these two haplotypes. As shown in **Figure 5B**, Hap2 exhibited significantly lower seed germination values compared to Hap1, suggesting that Hap 2 is the favorable allele of S*bPP2C33* for seed germination rate (**Figure 5B**).

### Metabolome analysis

**Figure 6:**
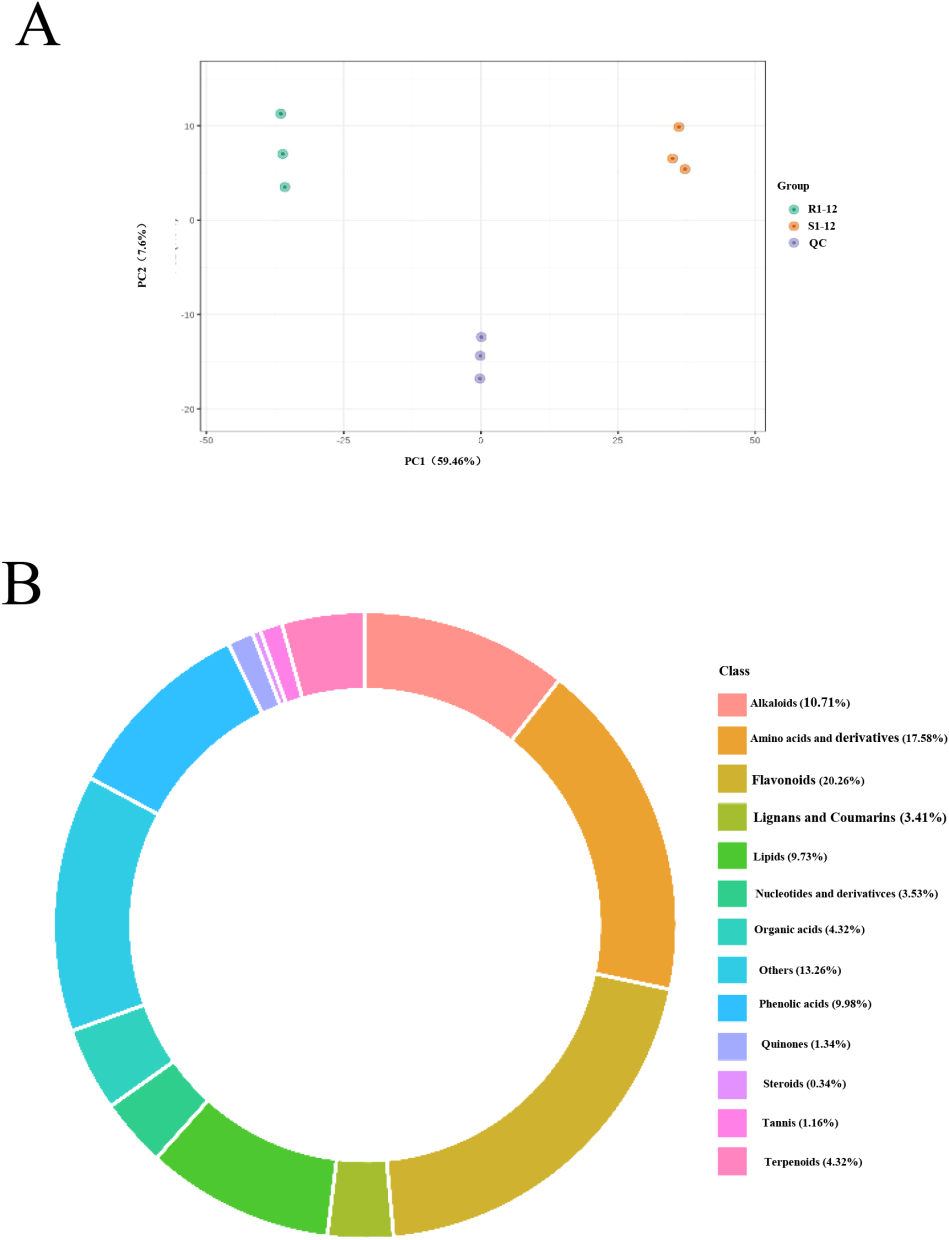
Metabolomic analysis of R1 and S1 at 24 h after seed germination. **(A)** Principal component analysis (PCA) of the metabolomic profiles of the six analyzed samples. **(B)** The categories of the identified metabolism. R1 and S1 represent TB kangya and Zhuyeqing (Kaiyuan), respectively.

Since phenotypic difference in seed germination is greatest at 24 h after seed germination and DEGs were enriched mainly in metabolic pathways and biosynthesis of secondary metabolites, R1–24 and S1–24 were used for further metabolome analysis. A total of 1,644 metabolites were identified, and PCA revealed the segregation of two samples (**Figure 6A**). These metabolites were mainly classified into 13 categories: alkaloids, amino acids and derivatives, flavonoids, lignans and coumarins, lipids, nucleotides and derivatives, organic acids, phenolic acids, quinones, steroids, tannins, terpenoids, and others (**Figure 6B**). Overall, 737 differentially expressed metabolites (DEMs) were screened (**Supplementary Table S9**).

### Starch and sucrose metabolism-related DEGs and DEMs to seed germination

**Figure 7:**
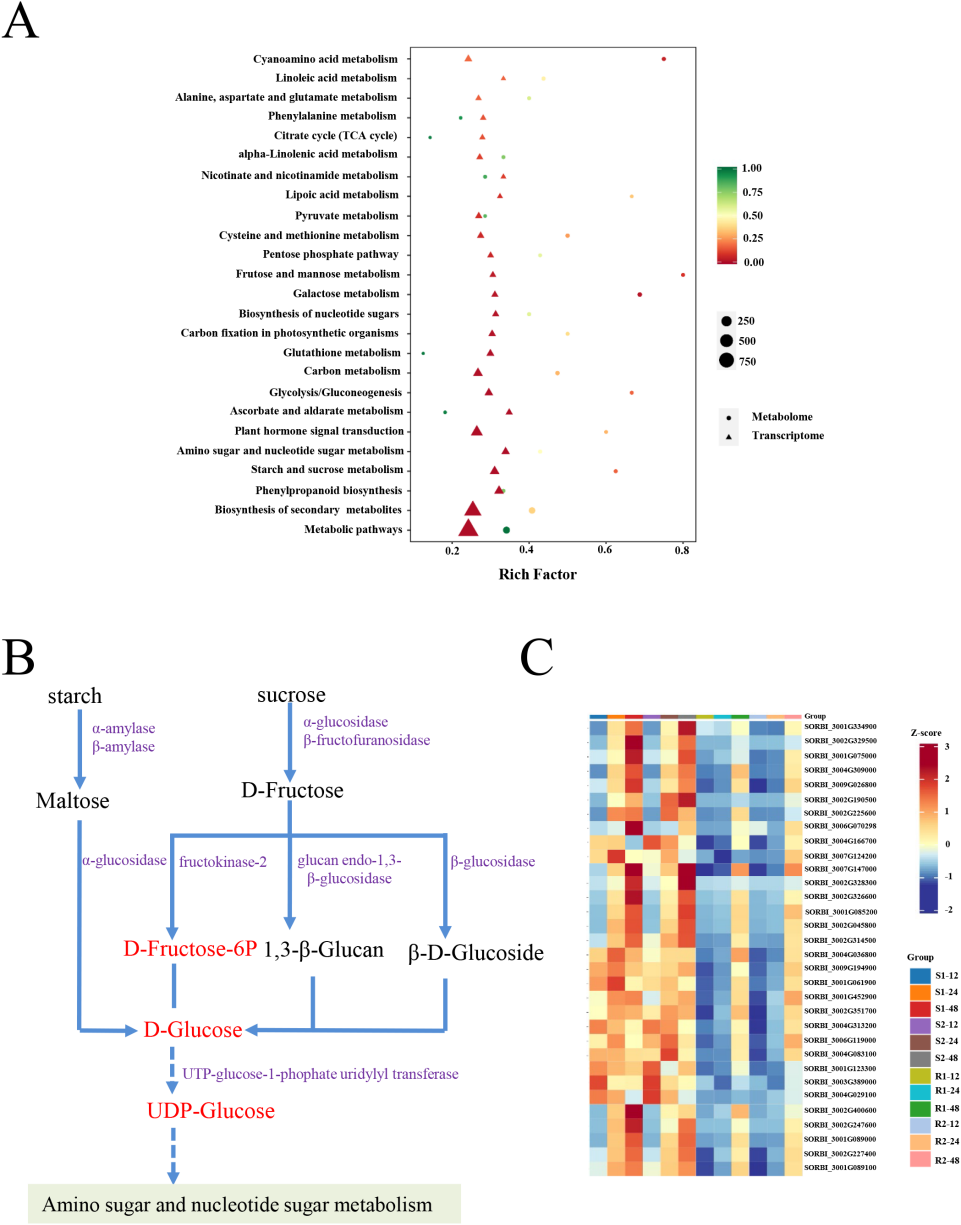
RNA-seq and metabolome combined analysis. **(A)** KEGG pathway enrichment of DEGs and DEMs. **(B)** The DEGs and DEMs of starch and sucrose metabolisms. The metabolites in **(B)** stained with red indicate up-accumulated, and the genes in **(B)** stained with purple indicate upregulated. **(C)** Heatmap analysis of genes involved in starch and sucrose metabolisms. R1 and R2 represent materials with higher resistance to germination, TB kangya and Jingnong2B, respectively. S1 and S2 represent materials with lower resistance to germination, Zhuyeqing (Kaiyuan) and Heiwobai (luanping), respectively.

Based on the transcriptome and metabolomic data in R1 and S1 at 24 h after seed germination, the relationships between DEGs and DRMs in different samples were systematically explored. KEGG pathway enrichment analysis based on transcriptome and metabolomic data revealed that DEGs and DEMs were also commonly enriched in starch and sucrose metabolism (**Figure 7A**). Many DEGs and DEMs were upregulated in materials with lower resistance to germination (S1, and S2) compared to materials with higher resistance to germination (R1 and R2). The expression of starch and sucrose degradation-related genes (α/β-amylase, α/β-glucosidase, β-fructofuranosidase, glucan endo-1,3-β-glucosidase, and UTP-glucose-1-phosphate uridylyltransferase) was significantly upregulated in four comparison groups (S1/R1, S1/R2, S2/R1, and S2/R2) (**Figures 7B, C**; **Supplementary Table S10**). Consistently, the metabolites in starch and sucrose metabolism (D-Fructose-6P, D-Glucose, and UDP-Glucose) were also accumulated (**Figure 7B**). Therefore, we suggested that the upregulation of these genes promotes the degradation of starch and sucrose and ultimately stimulates seed germination.

In the ABA signaling pathway, ABSCISIC ACID INSENSITIVE5 (ABI5), a master transcription factor in ABA signaling, binds to the ABA-responsive element (ABRE) of the promoter of target genes (such as *EARLY METHIONINE-LABELED 1* and *EARLY METHIONINE-LABELED 6*) to regulate their expression and ultimately inhibit seed germination (Carles et al., 2002). In our study, we found that the expression level of *ABI5* upregulated in materials with lower resistance to germination (S1 and S2) compared to materials with higher resistance to germination. Meanwhile, starch and sucrose metabolism is also essential for sorghum seed germination. For these reasons, we wondered whether starch and sucrose degradation-related DEGs were regulated by ABI5. To this end, we screened for ABRE motifs (consensus: ACGTG/G-box) in the promoters of DEGs associated with these pathways. As a result, five key DEGs harbored ≥2 ABREs within their promoter regions (≤2 kb upstream of TSS), suggesting potential direct transcriptional regulation by ABI5 (**Supplementary Figure S9**).

## Discussion

During the domestication of sorghum, to shorten the growth cycle during sorghum seed production, selection pressure favored less-dormant genotypes compared with wild ancestors to ensure rapid synchronous germination after sowing. However, the low dormancy of sorghum may lead to PHS, causing economic losses (Benech-Arnold and Rodríguez, 2018). Consequently, efforts have been directed to understand the molecular mechanism of seed dormancy and germination to cope with this situation.

In this study, we analyzed the germination phenotype in four sorghum lines and found that they showed contrasting germination abilities: S1 and S2 showed lower resistance to germination; however, R1 and R2 showed higher resistance to germination (**Figure 1**). We then performed a comprehensive analysis of the transcriptome profile of these four sorghum materials at three developmental stages in four comparison groups (S1/R1, S1/R2, S2/R1, and S2/R2). In these four comparison groups, the KEGG analysis based on transcriptome data showed that metabolic pathways, biosynthesis of secondary metabolites, and starch and sucrose metabolism were commonly enriched in all three stages, while plant hormone signal transduction was greatly enriched at 12 h after seed germination, suggesting that these factors play key roles in regulating sorghum seed germination (**Figure 2**).

Previous studies have shown that many phytohormones regulate seed germination. It is widely recognized that GA and ABA are the major hormones that have antagonistic effects on the regulation of seed germination (Shu et al., 2016). In this study, we found that in the ABA pathway, PYL/PYR and PP2C (negative regulators in ABA signaling pathway) were upregulated and ABI5 (positive regulators in ABA signaling pathway) were downregulated in materials with lower resistance to germination (**Figure 3**). These results demonstrated that these ABA signaling pathway-related genes may play key roles in regulating sorghum seed germination.

Currently, GWAS is an efficient approach that has been widely used in dissecting the genetic loci controlling complex agronomic traits in crops. In this study, a GWAS of seed germination rate at 24 h trait in sorghum was performed using 232 sorghum cultivars. A total of four significant SNPs associated with seed germination rate were identified (**Figure 4A**). The most significant SNP was on chromosome 2 including 31 candidate genes (**Figure 4B**). Through functional annotation of these 31 candidate genes, we classified them into eight distinct categories: cell wall biosynthesis (four genes), cell cycle regulation (three genes), plant defense and stress response (nine genes), senescence regulation (one gene), temperature sensing (one gene), sugar transport (one gene), plant development including seed germination (three genes), and uncharacterized function (nine genes) (**Supplementary Table S7**). Transcriptome analysis revealed that six of these genes showed differential expression patterns across three developmental stages in four comparison groups (**Supplementary Table S8**). Among these six genes, one gene (*SbPP2C 33*) was functionally annotated to plant developmental processes, suggesting its potential role in regulating germination characteristics.

PP2C is a key negative regulator of the ABA signaling pathway and is critical in regulating seed germination according to our transcriptome data (**Figure 3**). Additionally, the homolog of *SbPP2C33*, *OsPP2C51*, is mainly expressed in seeds and can interact with the PYL family to transmit ABA signals to positively regulate rice seed germination (Bhatnagar et al., 2017). Therefore, among the six genes, *SbPP2C33* was the most promising candidate gene responsible for the seed germination ability. *SbPP2C33* is the 33rd member of the PP2C family in sorghum.

According to these significant SNPs in the promoter of *SbPP2C33*, we identified two major haplotypes (Hap1 and Hap2) in *SbPP2C33.* Hap1 was the most prevalent and Hap 2 is the favorable allele for low seed germination ability (**Figure 5**). Previous studies have demonstrated that PP2C positively regulates seed germination (Bhatnagar et al., 2017; Yu et al., 2020). However, they did not analyze natural variations of PP2C, limiting its direct application in breeding programs. In our study, we further analyzed natural variations in *SbPP2C33* and identified superior haplotypes, which can be directly utilized in PHS resistance breeding through molecular markers developed from the SNPs located in the promoter region of *SbPP2C33*.

**Figure 8:**
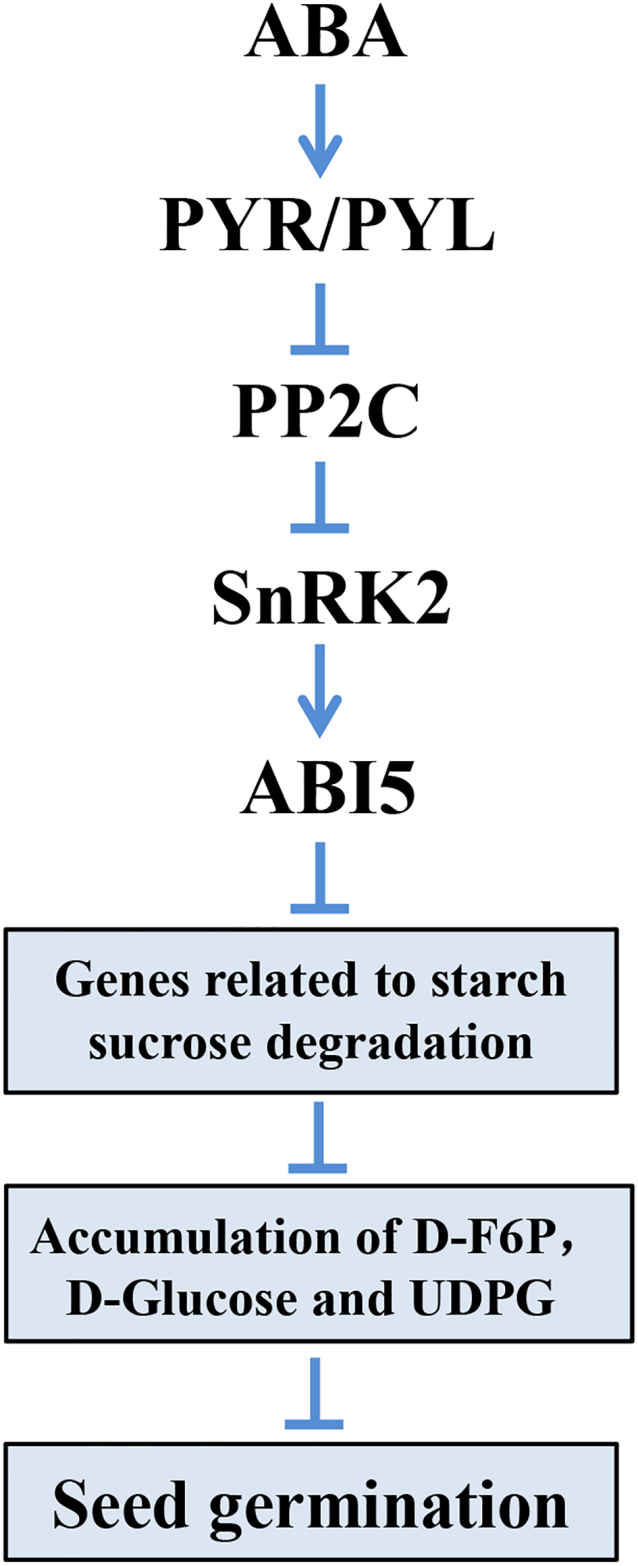
A proposed working model describing the mechanism of seed germination regulatory in sorghum.

In dormant seeds, metabolic activities are weak or silenced until when the dormancy was broken and germination began (Penfield, 2017). As sugars from starch hydrolysis are the major source of energy for seed germination (Beck and Ziegler, 1989), the related genes in starch and sucrose metabolism are essential. In our study, we found that genes encoding α-amylase, β-amylase, α-glucosidase, and β-fructofuranosidase were upregulated in materials with lower resistance to germination, indicating their roles in converting starch or sucrose to glucose and fructose, respectively (**Figure 7**). Also, related genes encoding α-glucosidase, β-glucosidase, fructokinase-2, glucan endo-1,3-β-glucosidase, and UTP-glucose-1-phophate uridylyltransferase that degrade maltose or fructose to glucose displayed similar patterns. Consistent with expression data, the metabolites in starch and sucrose metabolism (D-Fructose-6P, D-Glucose, and UDP-Glucose) were also accumulated (**Figure 7**). The results suggested that starch and sucrose metabolism provided the necessary energy for sorghum seed germination, and related genes are essential in this process. Furthermore, we found that five key DEGs related to starch and sucrose degradation harbored ≥ 2 ABREs within their promoter regions, suggesting potential direct transcriptional regulation by ABI5. In summary, we conclude that in seeds exhibiting high resistance to germination, the ABI5 protein, activated by ABA signaling, inhibits the expression of genes related to starch and sucrose degradation. This inhibition leads to decreased accumulation of D-F6P, D-Glucose, and UDPG, ultimately resulting in the suppression of seed germination (**Figure 8**).

Taken together, through integrated transcriptomic, metabolomic, and genome-wide association analyses, we revealed that ABA signaling coordinates carbohydrate mobilization during seed germination in sorghum. These findings provide novel mechanistic insights into the hormonal regulation of metabolic processes in cereal crops.

## Data Availability

All data supporting the findings of this study are available within the paper and within its **Supplementary Materials** published online. The raw sequence data reported in this paper have been deposited in the Genome Sequence Archive (Chen et al., 2021) in National Genomics Data Center (CNCB-NGDC Members and Partners, 2022), China National Center for Bioinformation/Beijing Institute of Genomics, Chinese Academy of Sciences (GSA: CRA025132) that are publicly accessible at https://ngdc.cncb.ac.cn/gsa.
