# Supplementary figures and images for: Integrated transcriptome, GWAS, and metabolome revealed the mechanism of seed germination in sorghum
Source: Front Plant Sci. 2025 Jul 17;16:1601899. doi: 10.3389/fpls.2025.1601899 (PMC12310604; doi:10.3389/fpls.2025.1601899)

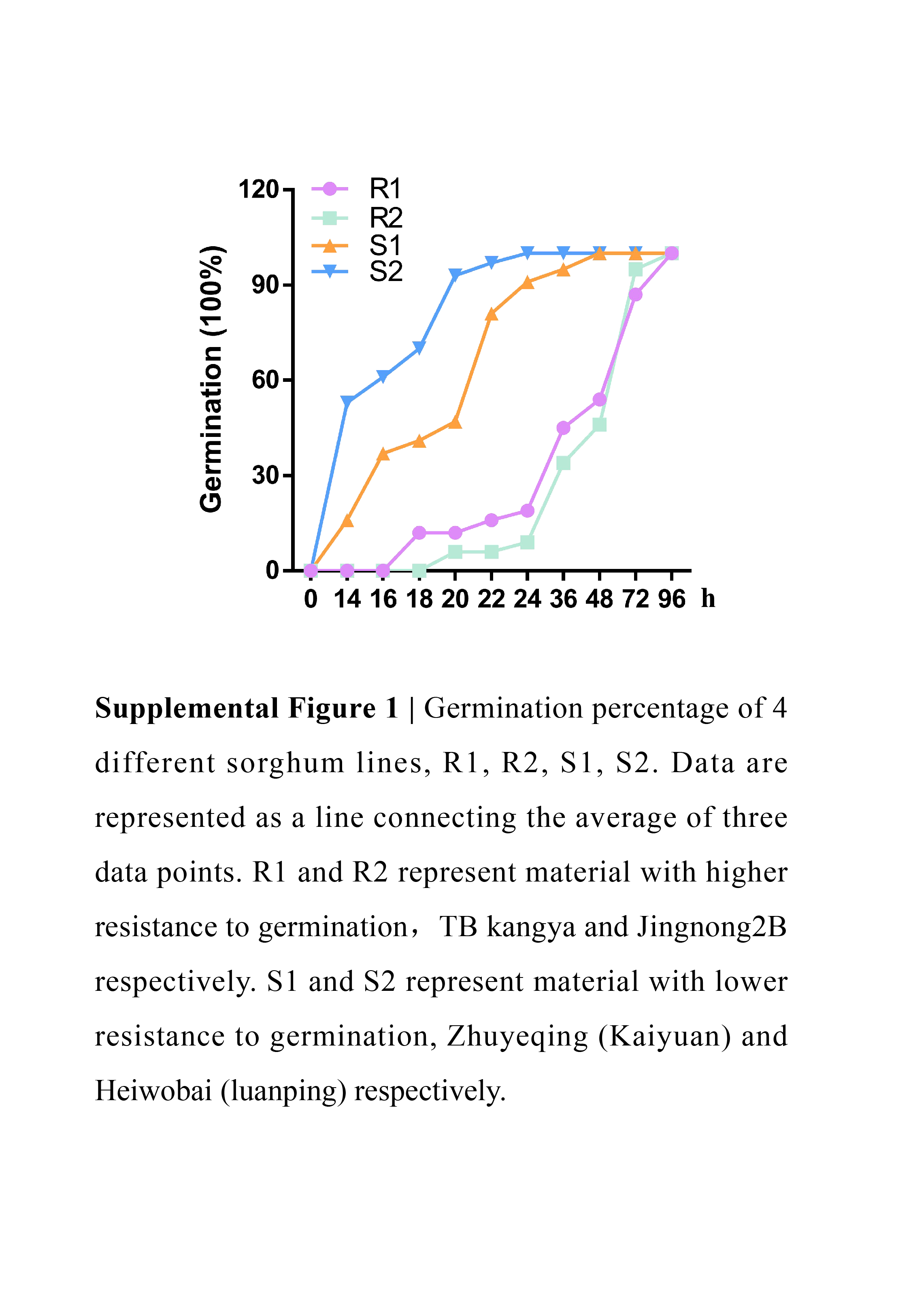

Supplement: Supplementary file 2 [file Image1.tif]

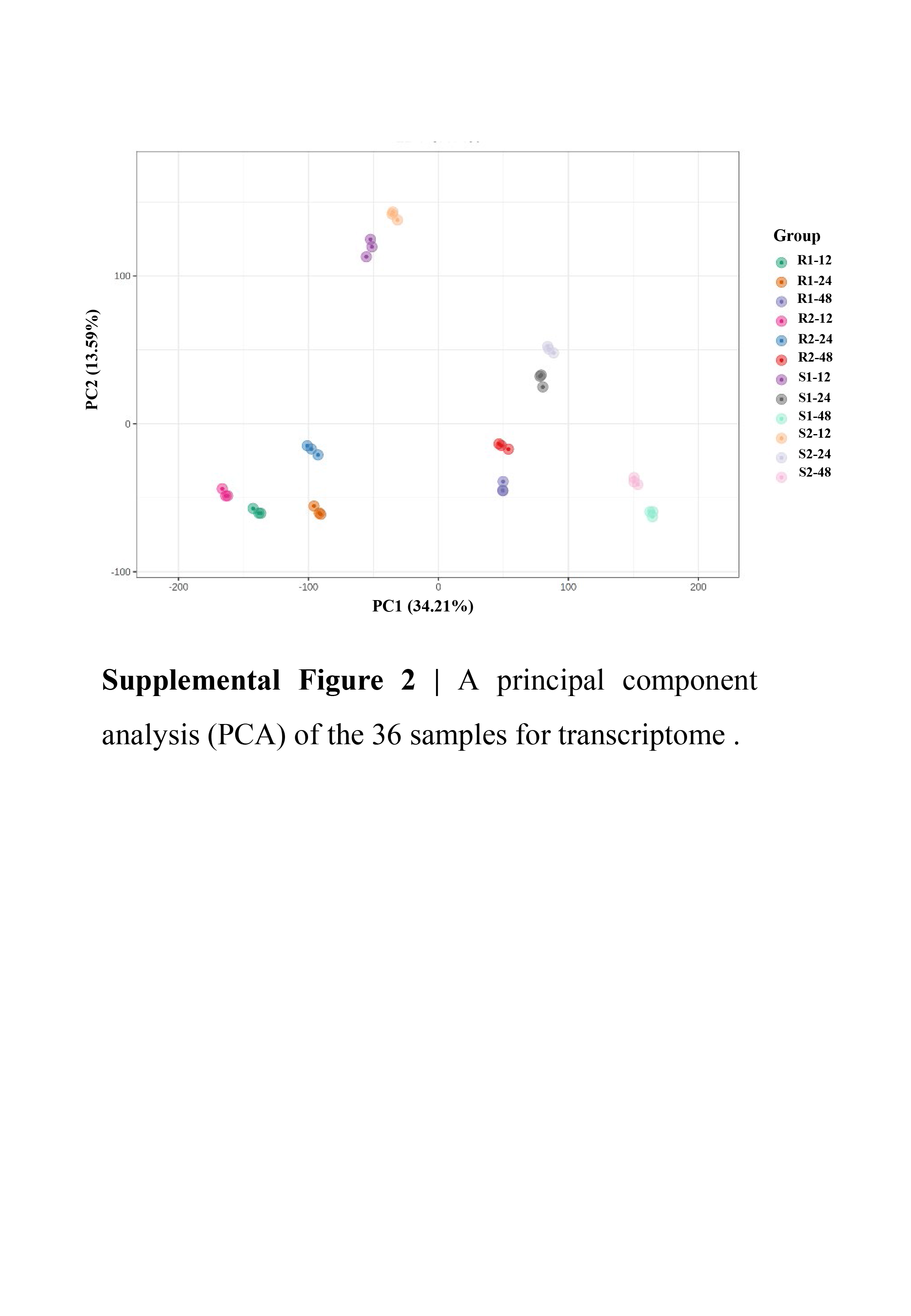

Supplement: Supplementary file 3 [file Image2.tif]

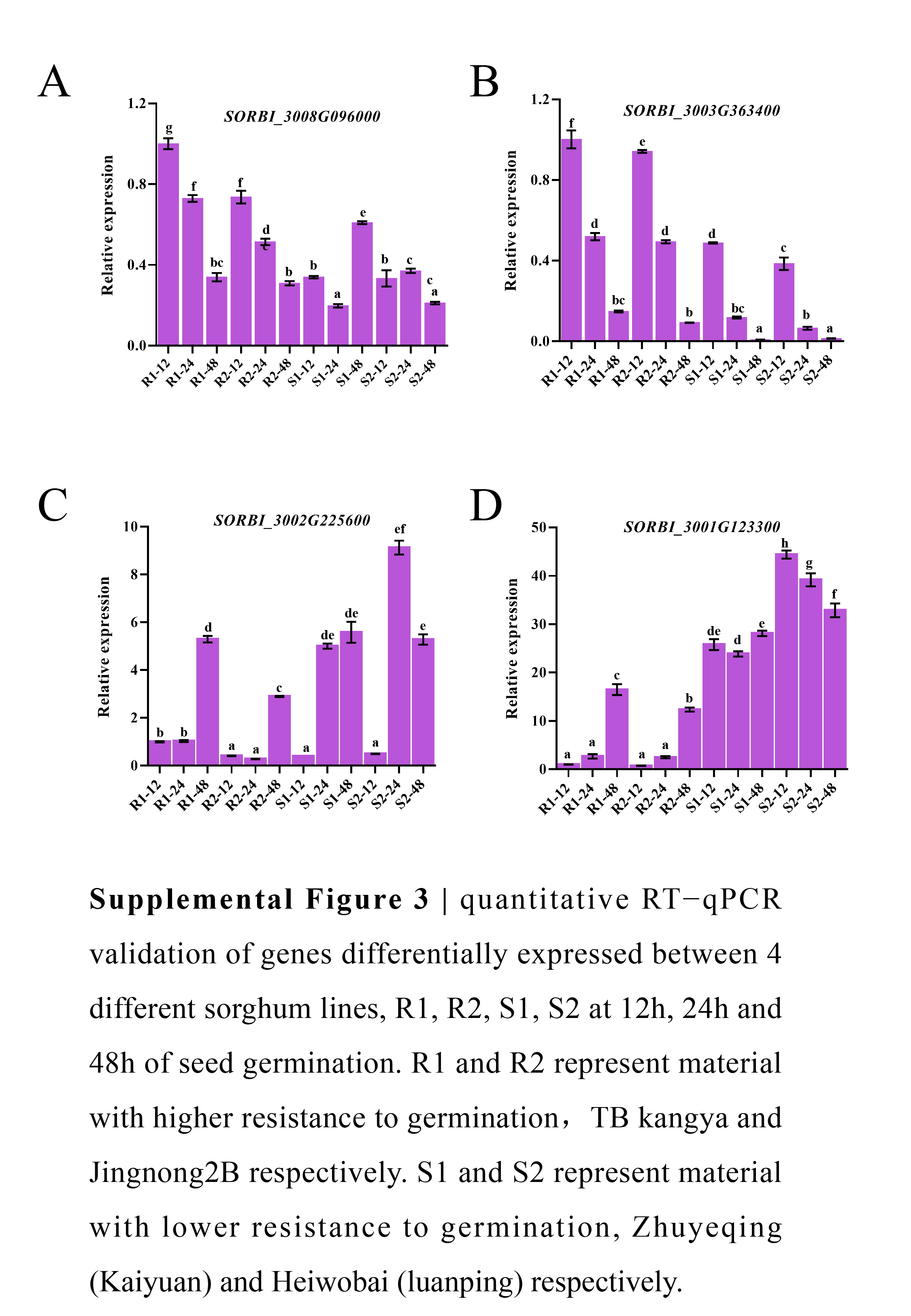

Supplement: Supplementary file 4 [file Image3.tif]

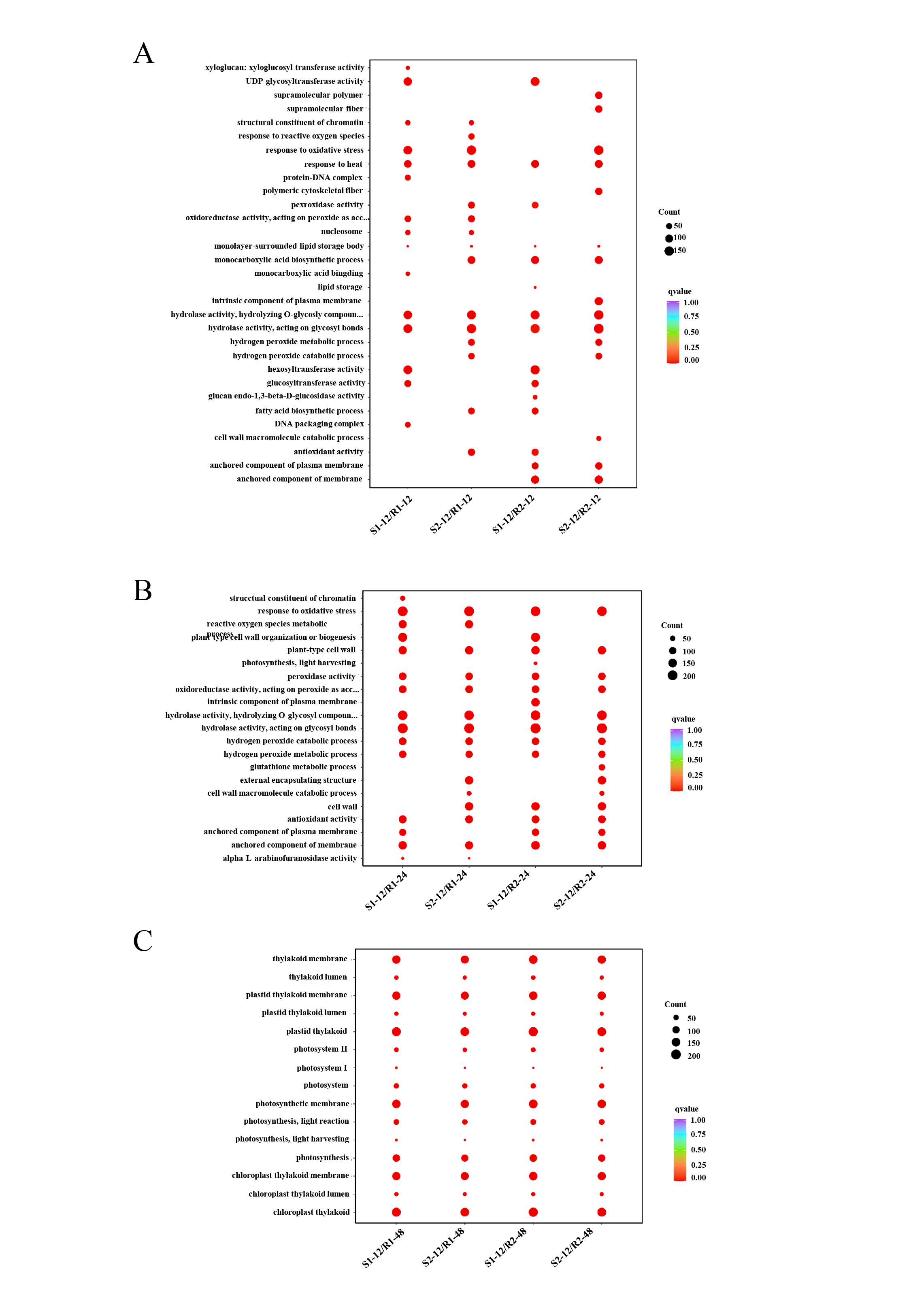

Supplement: Supplementary file 5 [file Image4.tif]

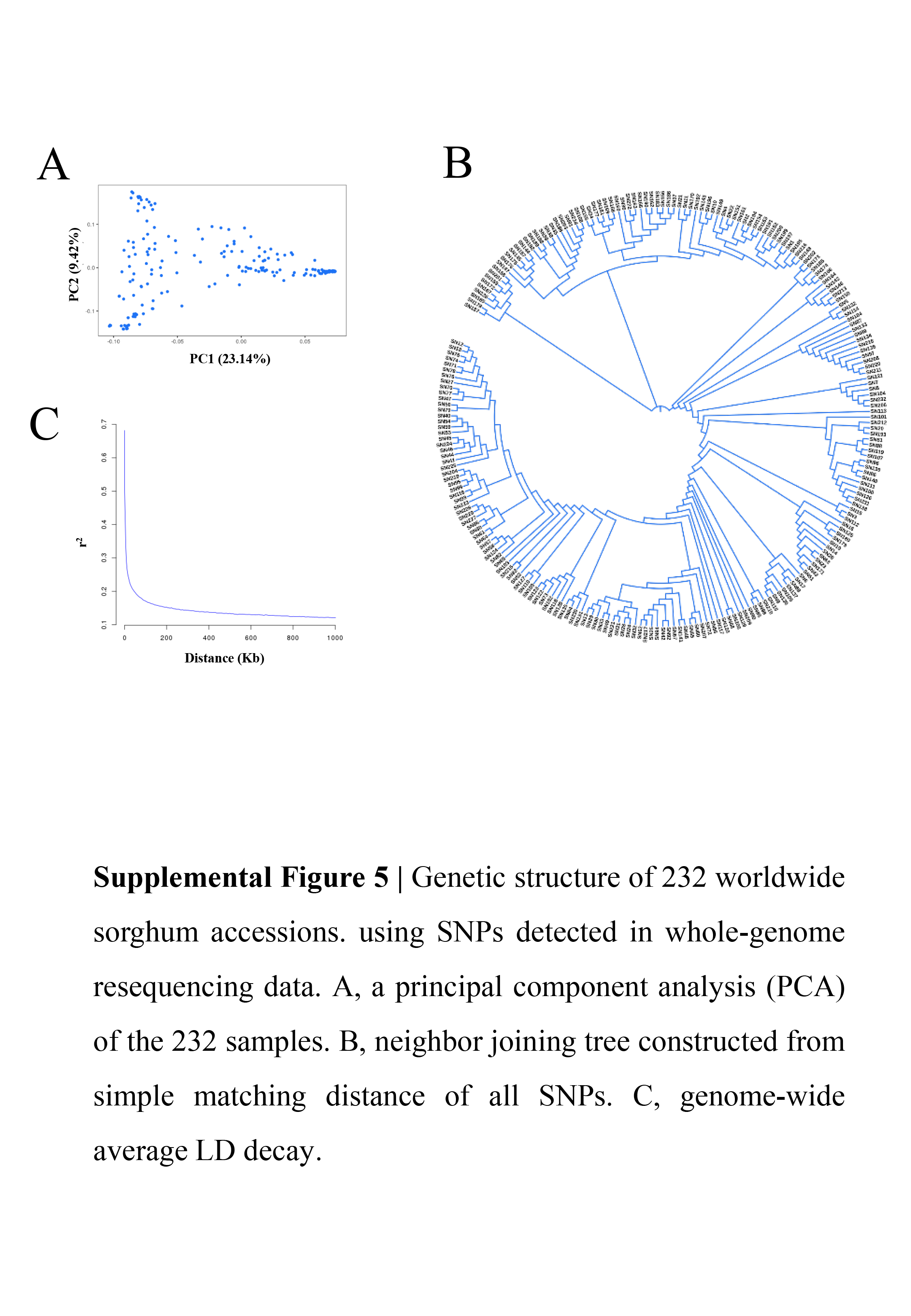

Supplement: Supplementary file 6 [file Image5.tif]

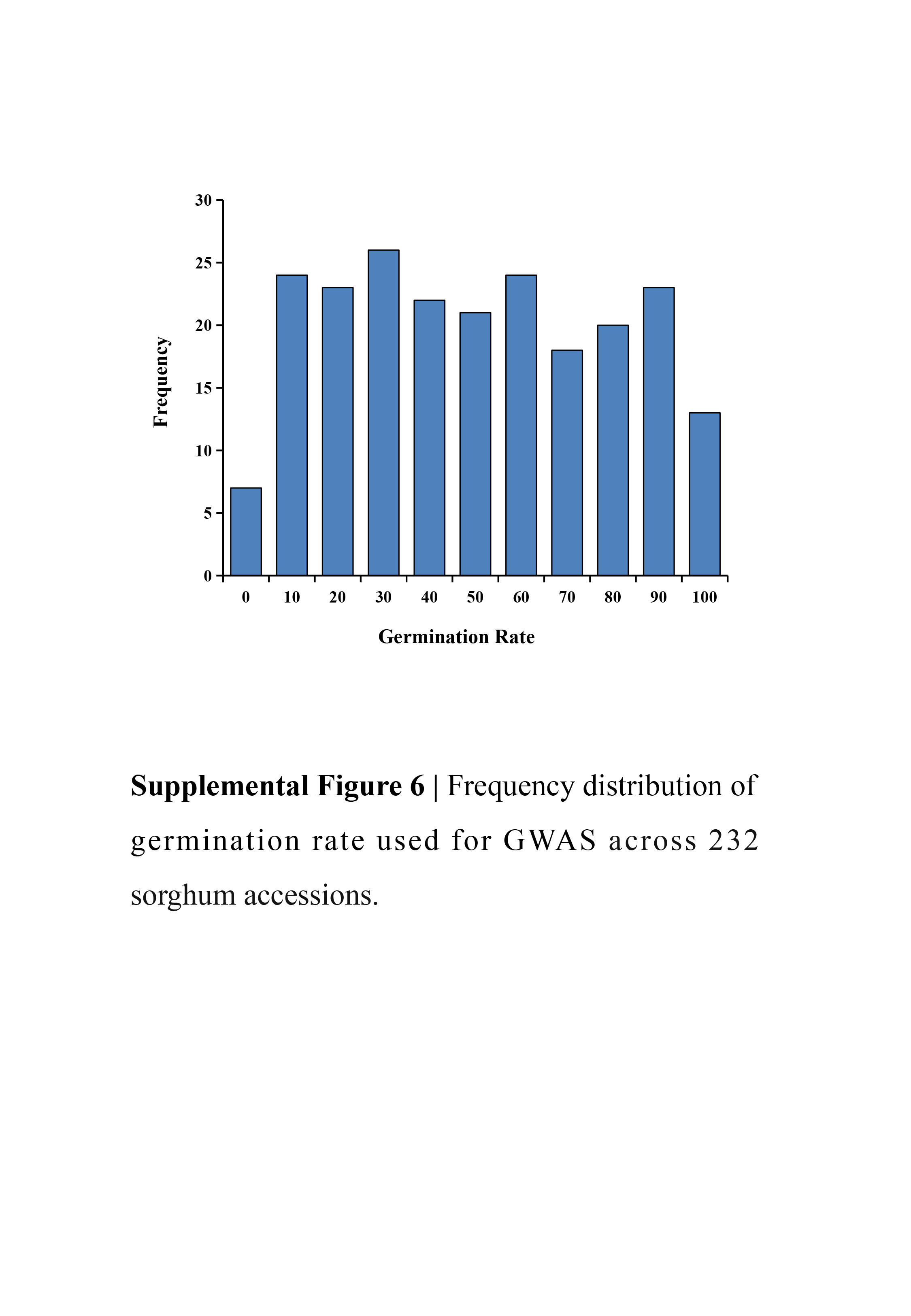

Supplement: Supplementary file 7 [file Image6.tif]

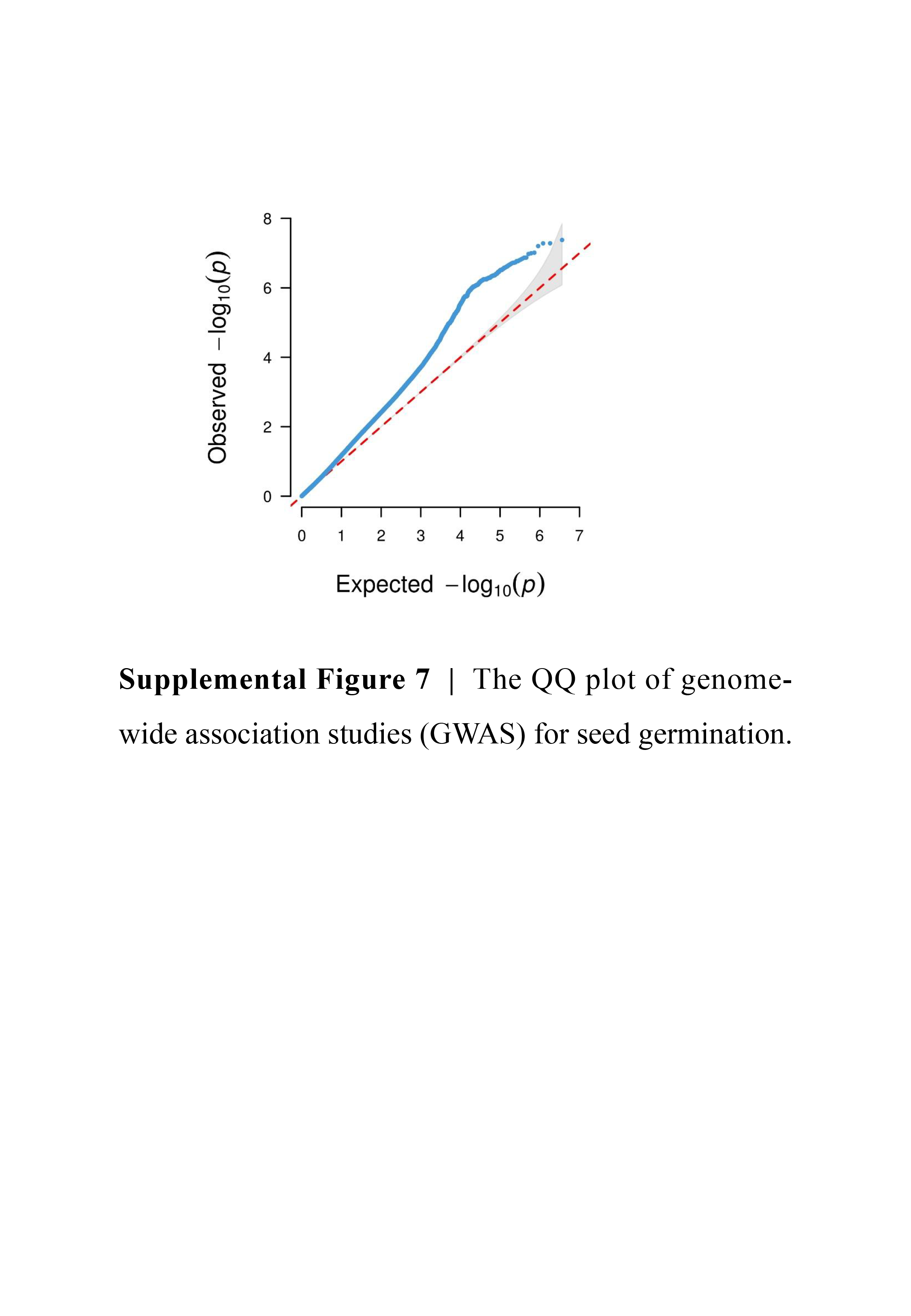

Supplement: Supplementary file 8 [file Image7.tif]

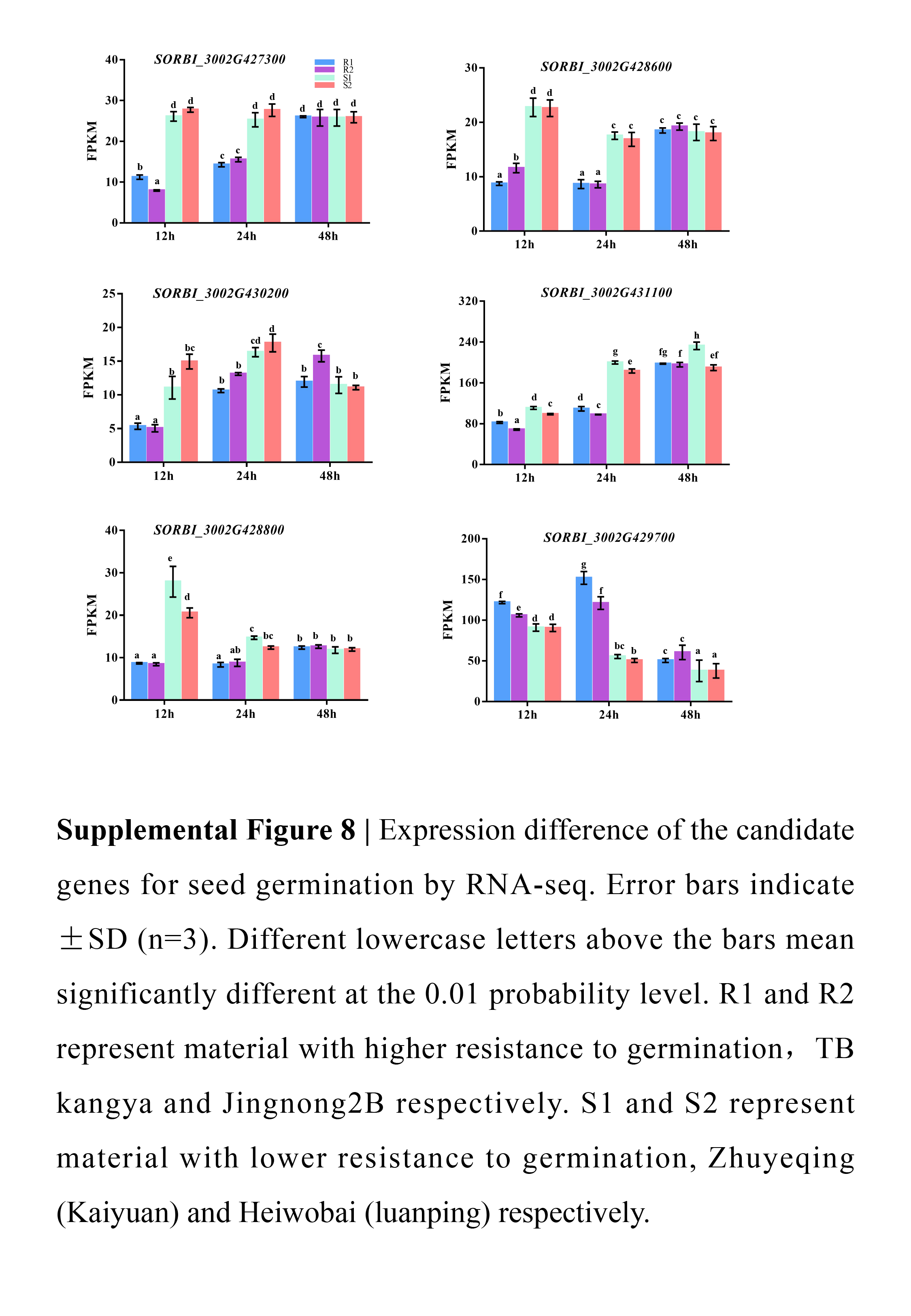

Supplement: Supplementary file 9 [file Image8.tif]

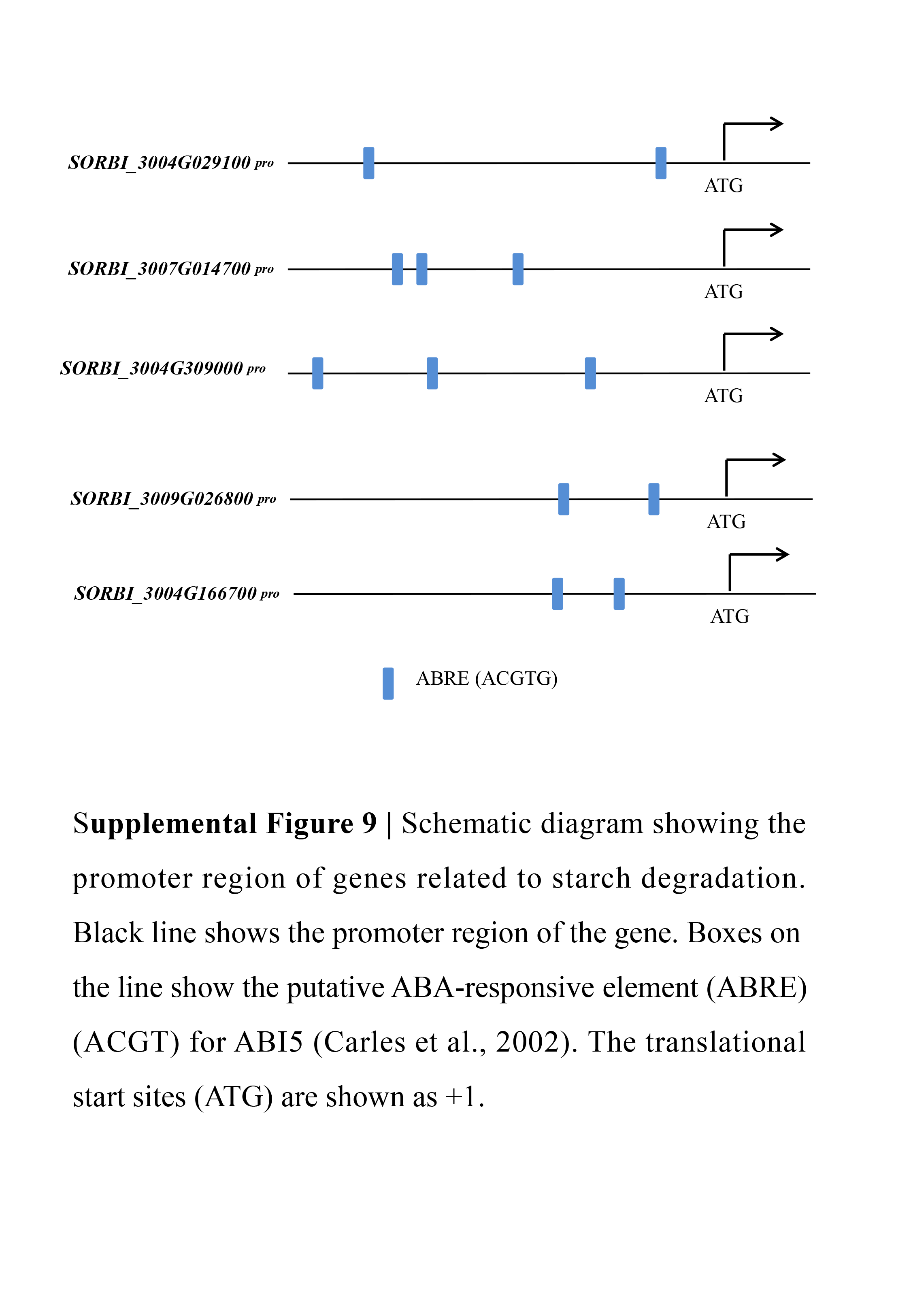

Supplement: Supplementary file 10 [file Image9.tif]
